# Determinants of low birth weight among neonates born in Amhara Regional State Referral Hospitals of Ethiopia: unmatched case control study

**DOI:** 10.1186/s13104-018-3568-2

**Published:** 2018-07-09

**Authors:** Getnet Asmare, Nigusie Berhan, Mengistu Berhanu, Animut Alebel

**Affiliations:** 1 College of Health Sciences, Debre Tabor University, Debre Tabor, Ethiopia; 2 College of Medicine and Health Sciences, University of Gondar, Gondar, Ethiopia; 3 College of Health Sciences, Debre Markos University, P. O. Box: 269, Debre Markos, Ethiopia

**Keywords:** Low birth weight, Maternal factors, Northwest Ethiopia

## Abstract

**Objective:**

This study was conducted to identify the determinants of low birth weight among infants born in Amhara Regional State Referral Hospitals of Ethiopia.

**Results:**

This study found that mothers who delivered female infants (AOR: 1.7, 95% CI 1.1, 2.6), occurrence of health problems during current pregnancy (AOR: 2.8, 95% CI 1.7,4.5), absence of antenatal care (AOR: 2.3,95% CI 1.3,4.0), lack of iron supplementation (AOR: 2.8, 95% CI 1.6,4.9), maternal MUAC below 23 cm (AOR: 1.7, 95% CI 1.0,2.7), and gestational age below 37 completed weeks (AOR: 3.3; 95% CI 1.9, 5.7) were found to be determinants of low birth weight.

## Introduction

Low birth weight (LBW) is defined as weight of the new born at birth less than 2500 g [1]. Globally, more than 20 million infants are born being low birth weight in each year. Of whom, majority of them were from developing countries, particularly Sub-Saharan countries including Ethiopia [2]. Africa is a home for 22% of low birth weight and in sub Saharan Africa low birth weight level is around 13–15% with a little variation across the regions [1]. According to the Ethiopian Demographic and Health Survey (EDHS 2011), the prevalence of low birth weight in Ethiopia is 11% [3]. Previous studies done in Ethiopia have shown that low birth weight prevalence was ranged from 22.5%, in Southwest and 17.5%, in Northwest [4, 5].

Low birth weight has both long and short-term complications unless early screening and interventions have been made [6]. Some of the long term complications of low birth weight include hypertension, diabetic nephropathy, proteinuria, progressive renal disease at late age, eye problems like strabismus and myopia, deafness, neurologic complications like cerebral palsy, developmental delay with IQ less than 70, epilepsy and behavioral disturbance [7]. The previous Ethiopian studies reported that different factors were significantly associated with low birth weight. Among these factors, being a female, first birth order and being a twin were significantly associated with low birth weight [5, 8]. Regarding maternal factors, maternal age at pregnancy, diet during pregnancy, her body composition at conception, lifestyle (alcohol, tobacco or drug abuse) exposure to malaria, HIV or syphilis or complications such as hypertension were also other factors significantly associated with low birth weight [9, 10]. Moreover, low socioeconomic status resulting in higher rates of maternal undernutrition, anemia, illness, inadequate prenatal care and obstetric complication has a strong positive correlation with low birth weight [11].

The Ethiopian government has acknowledged the severity of the problem, and currently some measures are taken by the government, Nongovernmental organizations (CU-ICAP, WHO), and professional associations like Ethiopian Pediatric Society [12, 13]. However, in Ethiopia only few researches have been conducted regarding the determinants of low birth weight, particularly in Amhara region. Therefore, this study aimed to identify the determinants of low birth weight focusing more of modifiable risk factors. The results of this study will be served as a baseline data for further studies. In addition it will be an input for planning health interventions to improve the wellbeing of children and women in Ethiopia, particularly in Amhara region.

## Main text

### Study area, design and period

An institution based unmatched case–control study was conducted among women who gave birth in Amhara region Referral Hospitals from March 20 to April 30, 2017. The region has a total of five referral hospitals. Of which, three of them were purposely selected because they have a large number of population in their catchment area (Debre Markos, Felege Hiwot, and University of Gondar referral hospitals). These three hospitals serve for more than 15 million populations in their catchment area.

### Population

All mothers who gave birth in Amhara Regional State Referral Hospitals were our source population. Mothers who gave live births weighed less than 2500 g were considered as cases and those mothers with live births weighed 2500 g and above were considered as controls. Mothers who had single birth infants were included. However, mothers who had infants with congenital anomaly and with chronic diseases (diabetes mellitus and hypertension) were excluded from the study. In addition, those mothers who were seriously ill during the data collection period, and those who were unable to communicate were excluded from the study.

### Sample size determination

The sample size was determined by using a double population proportion formula by considering the following statistical assumptions: 95% confidence interval (Zα/2 = 1.96), 80% power (Zβ = 0.84), case to control ratio 1:2 (r = 2), the odds ratio to be detected ≥ 2 and the 20% control group were exposed. The final sample size of the study was 453 (151 cases and 302 controls).

### Data collection procedures

Data were collected by using a structured interviewer administered questionnaire. The questionnaire was adapted from the Ethiopian Demographic and Health Survey. Trained midwives and nurses working outside the respective hospitals conducted the interviews and anthropometric measurements. The weight of the newborns was measured within 15 min after birth using a balanced scale. The scale was always checked and zeroed before weighing each newborn. Maternal height was measured against a wall height scale to the nearest centimeter. Patient records (charts) were also used to take some important variables like maternal hemoglobin level and co-morbid conditions.

### Data processing and analysis

Data were entered into Epi-data Version 3.1 and exported to SPSS version 22 for further analysis. Summary statistics (mean or median) for continues variables and percentage and frequency for categorical variables were computed for case and control groups separately. Both bivariable and Multivariable binary logistic regression were fitted for each explanatory variable. Finally, in the multivariable binary logistic regression analysis, adjusted odds ratio (AOR) with 95% CI and p-values were used to identify significant variables. Variables having p-value less than 0.05 were considered as significant determinants of low birth weight.

## Results

### Socio-demographic characteristics of the study participants

From a total of 453 sample size, 429 mothers-baby pairs (143 cases and 286 controls) were included in the final analysis making a response rate of (94.7%). More than half (55.2%) of mothers in cases and 40% in controls had female infants. The majority of mothers in cases and control group (72 and 84.3% respectively) were married. Regarding educational status, 35% of mothers among cases were unable to read and write, and 33.2% of mothers in the control group were diploma and above (Table 1).

**Table 1:** Sociodemographic characteristics of mothers and the newborns in Amhara Regional State Referral Hospitals, Ethiopia, 2017 (n = 429)

Variables	LBW		NBW		Total	
	Count (n)	Percent (%)	Count (n)	Percent (%)	Count (n)	Percent (%)
Infant sex						
Male	64	44.8	170	59.4	234	54.5
Female	79	55.2	116	40.6	195	45.5
Maternal age						
≤ 20	23	16.1	21	7.3	44	10.3
21–25	54	37.8	88	30.8	142	33.1
26–30	48	33.6	118	41.3	166	38.7
31–35	9	6.25	41	14.3	50	11.6
≥ 36	9	6.25	18	6.3	27	6.3
Marital status						
Married	103	72.0	241	84.3	344	80.2
Not married	4	2.8	11	3.8	15	3.5
Divorced	36	25.2	30	10.5	66	15.4
Widowed	0	0.0	4	1.4	4	0.9
Residence place						
Rural	72	50.3	103	36.0	175	40.8
Urban	71	49.7	183	64.0	254	59.2
Religion						
Orthodox	109	76.2	206	72.0	315	73.4
Muslim	31	21.7	57	19.9	88	20.5
Protestant	3	2.1	23	8.0	26	6.1
Occupational status						
Housewife	53	37.1	95	33.2	148	34.5
Merchant	22	15.4	56	19.6	78	18.2
Government employee	35	24.5	84	29.4	119	27.7
Farmer	26	18.2	38	13.3	64	14.9
Others	7	4.9	13	4.5	20	4.7
Average family monthly income						
≤ 1650	60	42.0	76	26.6	136	31.7
1651–3200	44	30.8	91	31.8	135	31.5
3201–5250	22	15.4	56	19.6	78	18.2
5251–7800	11	7.7	44	15.4	55	12.8
> 7800	6	4.2	19	6.6	25	5.8

### Maternal obstetrics and behavioral related factors

More than half (61.5%) of mothers in the case group and three quarters of mothers in the control group had a mid-upper arm circumference of below 23 cm. The majority of the mothers (83.2% in cases and 87.8% in controls) had no history of abortion (Table 2).

**Table 2:** Obstetrics and behavioral related history of mothers in Amhara Regional State Referral Hospitals, Ethiopia, 2017 (n = 429)

Variables	LBW		NBW		Total	
	Count (n)	%	Count (n)	%	Count (n)	%
MUAC						
< 23 cm	88	61.5	217	75.9	305	71.1
≥ 23 cm	55	38.5	69	24.1	124	28.9
History of abortions						
Yes	24	16.8	35	12.2	59	13.8
No	119	83.2	251	87.8	370	86.2
Mode of delivery						
Spontaneous vaginal	113	79.0	188	65.7	301	70.2
Assisted vaginal	3	2.1	22	7.7	25	5.8
Caesarean section	27	18.9	76	26.6	103	24.0
ANC visit						
Yes	101	70.6	245	85.6	346	80.7
No	42	29.4	41	14.4	83	19.3
Health problems						
Yes	77	53.8	111	38.8	188	43.8
No	66	46.2	175	61.2	241	56.2
Iron supplementation						
Yes	95	66.4	244	85.3	339	79.0
No	48	33.6	42	14.7	90	20.1
Gravidity						
Primi gravid	70	49.0	123	43.0	193	45.0
Multigravida	73	51.0	163	57.0	236	55.0
Parity						
Primi-para	79	55.2	129	45.1	208	48.5
Multipara	64	44.8	157	54.9	221	51.5
Gestational age						
Preterm	48	33.6	37	12.9	85	19.8
Term and above	95	66.4	249	87.1	344	80.2
History of low birth weight						
Yes	26	18.2	29	10.1	55	12.8
No	117	81.8	257	89.9	374	87.2
History of trauma						
Yes	17	4.9	14	11.9	31	7.2
No	125	94.1	269	87.4	398	92.8
Ever drink alcohol						
Yes	19	13.3	23	8	42	9.8
No	117	86.7	263	92	387	90.2
Ever chew chat						
Yes	3	2.1	1	0.3	4	0.9
No	140	97.9	285	99.7	425	99.1
Ever smoke cigarette						
Yes	0	0.0	0	0.0	0	0.0
No	143	100.0	286	100.0	429	100.0

### Determinants of low birth weight

In multivariable binary logistic regression analyses, mothers who encountered any pregnancy related problems during their current pregnancy were more prone to have a low birth weight baby as compared to mothers who didn’t encounter any health problems (AOR: 2.8, 95% CI 1., 4.5). The odds of low birth weight was higher among female neonates as compared to their male counterparts (AOR: 1.7, 95% CI 1.1, 2.6). The odds of low birth weight was also higher among mothers who didn’t attend ANC as compared to mothers who attended ANC follow up in the current pregnancy (AOR: 2.3, 95% CI 1.3, 4.0). The odds of low birth weight was also higher among mothers who did not take iron supplementation as compared to mothers who took iron supplementation during the current pregnancy (AOR: 2.8, 95% CI 1.6, 4.9). Mothers who had MUAC below 23 cm (AOR: 1.7, 95% CI 1.0, 2.7) and gestational age below 37 completed weeks (AOR: 3.3, 95% CI 1.95, 5.7) were found to be risk factors for low birth weight (Table 3).

**Table 3:** Association of factors with low birth weight in Amhara Regional State Referral Hospitals, Ethiopia, 2017 (n = 429)

Variables	LBW		LBW		COR	AOR
	(n)	(%)	(n)	(%)		
Sex						
Male	64	44.8	170	59.4	1	1
Female	*79*	*55.2*	*116*	*40.6*	*1.81* (*1.2, 2.7*)	*1.7* (*1.1, 2.6*)
Residence place						
Rural	72	50.3	103	36.0	1.8 (1.20–2.27)	1.0 (0.6, 1.8)
Urban	71	49.7	183	64.0	1	1
Educational status						
Unable to read and write	50	35.0	59	20.6	2.44 (1.41–4.21)	1.32 (0.69–2.52)
Grade 1–8	33	23.1	63	22.0	1.51 (0.85–2.69)	0.93 (0.48–1.79)
Grade 9–12	27	18.9	69	24.1	1.13 (0.62–2.04)	0.92 (0.48–1.76)
Diploma and above	33	23.1	95	33.2	1	1
MUAC category						
≥ 23 cm	88	61.5	217	75.9	1	1
< 23 cm	*55*	*38.5*	*69*	*24.1*	*1.97* (*1.28*–*3.03*)	*1.66* (*1.02*–*2.70*)
History of abortions						
Yes	24	16.8	35	12.2	1.45 (0.82–2.54)	1.38 (0.71–2.67)
No	119	83.2	251	87.8	1	1
ANC visit						
Yes	101	29.2	245	70.8	1	1
No	*42*	*50.6*	*41*	*49.4*	*2.49* (*1.52*–*4.05*)	*2.31* (*1.32*–*4.04*)
Complications during pregnancy						
Yes	*77*	*41.0*	*111*	*59.0*	*1.84* (*1.23*–*2.76*)	*2.79* (*1.74*–*4.45*)
No	66	27.4	175	72.6	1	1
Iron tabs given						
Yes	95	66.4	244	85.3	1	1
No	*48*	*33.6*	*42*	*14.7*	*2.94* (*1.82*–*4.73*)	*2.82* (*1.62*–*4.91*)
Parity						
Primi-para	79	55.2	129	45.1	1.5 (1.03–2.25)	1.45 (0.92–2.31)
Multipara	64	44.8	157	54.9	1	1
Gestational age						
Preterm	*48*	*56.5*	*37*	*43.5*	*3.4* (*2.08*–*5.55*)	*3.33* (*1.95*–*5.67*)
Term and above	95	27.6	249	72.4	1	1
History of LBW						
Yes	26	18.2	29	10.1	1.97 (1.11–3.49)	1.85 (0.97–3.35)
No	117	81.8	257	89.9	1	1

Italic values indicates significantly associated in the multivariable analysis

## Discussion

In this study, we aimed to identify the determinants of low birth weight among mothers who gave birth in Amhara region referral hospitals, Northwest Ethiopia. The findings of this study revealed that newborn characteristic such as sex was found to be significantly associated with low birth weight. Accordingly, the risk of low birth weight was higher among female neonates as compared to their male counterparts. This finding is consistent with the findings of earlier studies conducted in Ethiopia and Nigeria [11, 14]. In the present study, Nutritional status of women as proxy by MUAC was also found to be a significant determinate of LBW. This finding is consistent with a study conducted at Kersa, Ethiopia [15]. The nutritional status of the newborns ultimately depends on the nutritional status of the mothers during the time of pregnancy because the baby solely depends on placental feeding throughout the entire pregnancy.

Moreover, the study found that mothers who encountered pregnancy related problems during their current pregnancy were at higher risk to deliver low birth weight baby than mothers who didn’t have complications. This finding is similar to studies conducted in Tigray, Northern Ethiopia and Bale zone, Southeast Ethiopia [4, 16]. Mothers who had pregnancy related complications like preeclampsia are at higher risk of low birth weight than mothers who didn’t have complications. This is because of most commonly women with preeclampsia or pregnancy related hypertensive disorders end up with abruptio placenta this results decreasing nutrition and perfusion to the fetus finally end up with low birth weight or fetal death.

The risk of having low birth weight baby was higher among mothers who didn’t attend antenatal care in their current pregnancy as compared to mothers who attended ANC. Different studies done in different counters also supported this finding as birth weight was significantly associated with ANC service utilization [2, 4, 5, 8, 15]. Antenatal care visits are very important for both newborns and mothers as they provide chances for timely detection and intervention of feto-maternal problems and enable the mother to promote her health through counseling that she might receive. Another possible explanation might be mothers who had ANC follow up could get nutritional counseling to improve heir dietary diversity that enables her and her fetus for better pregnancy outcome.

Likewise, mothers who didn’t get iron supplementation were also more risk to deliver low birth weight infant than mothers who took iron supplementation during the current pregnancy. This supported with a study done in Kerala state, India [17]. Iron and folic acid supplementation for pregnant mothers has a great importance to prevent anemia during pregnancy, thereby enhancing better health outcome for both the mother and the fetus [18].

Furthermore, in this study, we also found that preterm (gestational age below 37 completed weeks) was found to be a risk factor for LBW. Supportive finding were obtained from studies done in Bale zone, Southeast Ethiopia [4] and Tigray region, Northern Ethiopia [19]. It is well known that as the gestational age of the fetus falls below, the term level the body weight of the fetus falls dramatically due to prematurity.

This study found that infant sex being female, preterm, absence of ANC visits, MUAC less than 23 cm, lack of iron or folic acid supplementation and complication during pregnancy the current pregnancy were found to be significant determinants of low birth weight.

## Limitations

Since the majority of cases were referred from other health institutions, variables like pre-pregnancy weight and gestational age of the first ANC visit were difficult to access. Therefore, these variables were not addressed in this study. In addition, important variables like physical activity and exposure of ambient air pollution were not assessed because we adapted the EDHS tool, which had no such components.

## Abbreviations

ANC: antenatal care
EDHS: Ethiopia Demographic and Health Survey
LBW: low birth weight
MUAC: mid upper arm circumference
NBW: normal birth weight
WHO: World Health Organization

## References

[1] Lawn JE, Gravett MG, Nunes TM, Rubens CE, Stanton C (2010). Global report on preterm birth and stillbirth (1 of 7): definitions, description of the burden and opportunities to improve data. BMC Pregnancy Childbirth.

[2] Mumbare SS, Maindarkar G, Darade R, Yenge S, Tolani MK, Patole K (2012). Maternal risk factors associated with term low birth weight neonates: a matched-pair case control study. Indian Pediatr.

[3] Demographic E: Health Survey: Addis Ababa. Ethiopia and Calverton, Maryland, USA: Central Statistics Agency and ORC Macro; 2011.

[4] Demelash H, Motbainor A, Nigatu D, Gashaw K, Melese A (2015). Risk factors for low birth weight in Bale zone hospitals, South-East Ethiopia: a case-control study. BMC Pregnancy Childbirth.

[5] Zeleke BM, Zelalem M, Mohammed N (2012). Incidence and correlates of low birth weight at a referral hospital in Northwest Ethiopia. Pan Afr Med J.

[6] Al Hazzani F, Al-Alaiyan S, Hassanein J, Khadawardi E (2011). Short-term outcome of very low-birth-weight infants in a tertiary care hospital in Saudi Arabia. Ann Saudi Med.

[7] Hack M, Klein NK, Taylor HG (1995). Long-term developmental outcomes of low birth weight infants. Fut Child.

[8] Mahumud RA, Sultana M, Sarker AR (2017). Distribution and determinants of low birth weight in developing countries. J Prev Med Public Health Yebang Uihakhoe chi.

[9] Dharmalingam A, Navaneetham K, Krishnakumar CS (2010). Nutritional status of mothers and low birth weight in India. Matern Child Health J.

[10] Pfinder M (2014). Anthropometric and health-related behavioral factors in the explanation of social inequalities in low birth weight in children with prenatal alcohol exposure. Int J Environ Res Public Health.

[11] Bugssa G, Dimtsu B, Alemayehu M (2014). Socio demographic and maternal determinants of low birth weight at Mekelle Hospital, Northern Ethiopia: a cross sectional study. Am J Adv Drug Deliv.

[12] The Federal Democratic Republic of Ethiopia Ministry of Health. National newborn and child survival strategy document brief summary 2015/16-2019/20. In: FMOH/MCH, Editor. Directorate. Addis ababa; 2015.

[13] Fulton C (2013). Improving neonatal mortality in an Ethiopian referral hospital. BMJ Qual Improv Rep.

[14] Muchemi OM, Echoka E, Makokha A (2015). Factors associated with low birth weight among neonates born at Olkalou District Hospital, Central Region, Kenya. Pan Afr Med J.

[15] Assefa N, Berhane Y, Worku A (2012). Wealth status, mid upper arm circumference (MUAC) and antenatal care (ANC) are determinants for low birth weight in Kersa, Ethiopia. PLoS ONE.

[16] Gebremedhin M, Ambaw F, Admassu E, Berhane H (2015). Maternal associated factors of low birth weight: a hospital based cross-sectional mixed study in Tigray, Northern Ethiopia. BMC Pregnancy Childbirth.

[17] Ismail IM, Venugopalan P (2016). Case-control study on risk factors of low birth weight in a tertiary care hospital, Kerala. Ann Community Health.

[18] Abu-Ouf NM, Jan MM (2015). The impact of maternal iron deficiency and iron deficiency anemia on child’s health. Saudi Med J.

[19] Gebremedhin M, Ambaw F, Admassu E, Berhane H (2015). Maternal associated factors of low birth weight: a hospital based cross-sectional mixed study in Tigray, Northern Ethiopia. BMC Pregnancy Childbirth.

